# Bibliometric and visualized analysis of the therapeutic application of suprachoroidal space from 2000 to 2024

**DOI:** 10.1097/JS9.0000000000003384

**Published:** 2025-09-08

**Authors:** Kehan Jin, Lihui Meng, Jingyuan Yang, Chan Zhao, Youxin Chen

**Affiliations:** a Department of Ophthalmology, Peking Union Medical College Hospital, Chinese Academy of Medical Sciences, Beijing, China; b Key Laboratory of Ocular Fundus Diseases, Chinese Academy of Medical Sciences & Peking Union Medical College, Beijing, China; c Beijing Key Laboratory of Fundus Diseases Intelligent Diagnosis & Drug/Device Development and Translation, Beijing 100730, China

**Keywords:** bibliometrics, drug delivery, microneedles, minimally invasive glaucoma surgery, retinal prosthesis, suprachoroidal space

## Abstract

**Objectives:**

To provide a bibliometric overview of the global research on the therapeutic applications of the suprachoroidal space (SCS) from 2000 to 2024.

**Methods:**

Publications were retrieved from the Web of Science Core Collection using a defined search strategy. A total of 776 articles were analyzed for trends in publication volume, countries, institutions, authorship, journals, citations, and keywords. An analysis of top 10% highly cited publications was also conducted. Co-authorship, co-occurrence, and citation burst analyses were conducted using VOSviewer, bibliometrix (R), and CiteSpace.

**Results:**

Global publications on SCS therapy increased steadily, with a 575% growth. The United States led in output (n = 274, 35.3%) and international collaboration. Top institutions included the University of Melbourne, Bionics Institute, and Osaka University. Fujikado Takashi was the most prolific author (34 publications), while Prausnitz Mark R R had the highest citation impact (50 per article). *Investigative Ophthalmology & Visual Science* was the most active journal (n = 50; 2249 citations). Keyword analysis revealed three main clusters: (1) drug delivery, (2) glaucoma treatment, and (3) retinal prosthesis. Overlay map indicated that research foci have shift from implantation to injection, minimally invasive approach, and clinical trials. Burst keywords showed suprachoroidal drug delivery (including gene therapy, ranibizumab, and triamcinolone acetonide) was a rapidly evolving and promising field for innovation, which was confirmed by analysis of high-impact publications.

**Conclusions:**

This study maps the global research landscape and emerging trends on the therapeutic applications of the SCS, underscores its translational nature, and fosters interdisciplinary collaboration in this evolving field.

**HIGHLIGHTS**

- This study is the first bibliometric analysis focused on the therapeutic applications of the suprachoroidal space (SCS).
- Annual publications related to SCS have increased by over 500% since 2000. The United States, Australia, Germany, and Japan are among the leading contributors.
- The research trend is gradually shifting from implants to microneedle injection, gene/drug delivery, and macular diseases.
- Microneedle-based SCS delivery offers a safe and clinically promising approach, with ongoing trials supporting its efficacy across multiple retinal diseases.

## Introduction

The suprachoroidal space (SCS) is a potential space between the sclera and the choroid in the eye. Anatomically, it is a narrow, fluid-filled compartment that extends circumferentially around the eye, forming part of the uveoscleral outflow pathway. Although not visible under normal physiological conditions, the SCS can be expanded by agents like viscoelastic or devices^[1,2]^, making it accessible for therapeutic interventions. Its proximity to the choroid and retina allows for a direct access to the posterior segment while minimizing exposure to anterior ocular tissues. Therefore, it has garnered increasing attention in recent years. This space serves as a promising route for targeted drug delivery and minimally invasive surgical interventions^[3,4]^, making it an ideal site for treating conditions such as uveitis, macular degeneration, and glaucoma^[5–7]^. Moreover, the development of innovative technologies, such as microneedles[2], nanoparticles^[8,9]^, controlled-release preparation[10], and suprachoroidal implants[11], has expanded the potential applications of the SCS in both clinical and experimental settings. Beyond its use in drug delivery, the SCS has also been explored in areas like retinal prostheses and suprachoroidal-transretinal stimulation (STS), offering hope for patients with degenerative retinal diseases such as retinitis pigmentosa[12]. Despite the growing interest in the SCS, a comprehensive understanding and visualization of its applications and research trends remains limited.

Bibliometric analysis is a powerful method for quantitatively evaluating the research landscape within a specific field[13]. By analyzing publication data, including keywords, citation patterns, and collaborative networks, bibliometric studies provide insights into the evolution of research trends, influential topics, and key contributors. This approach has been widely used in various medical specialties to identify emerging areas of interest and guide future research directions.

This study aims to provide a comprehensive bibliometric analysis of publications related to the treatment applications of SCS from 2000 to 2024, using data extracted from the Web of Science Core Collection (WoSCC). Further discussions were conducted into three important subtopics identified by bibliometric characterization, as well as challenges encountered and future perspectives. The findings of this study will not only enhance our understanding of the current status of the research landscape but also provide valuable insights to guide future investigations and clinical applications involving the SCS.

This study adheres to the TITAN Guidelines 2025 for transparent and responsible use of AI[14]. No AI technologies were used in the generation, analysis, or writing of this manuscript.

## Materials and methods

### Search strategy and data collection

**Figure 1.**
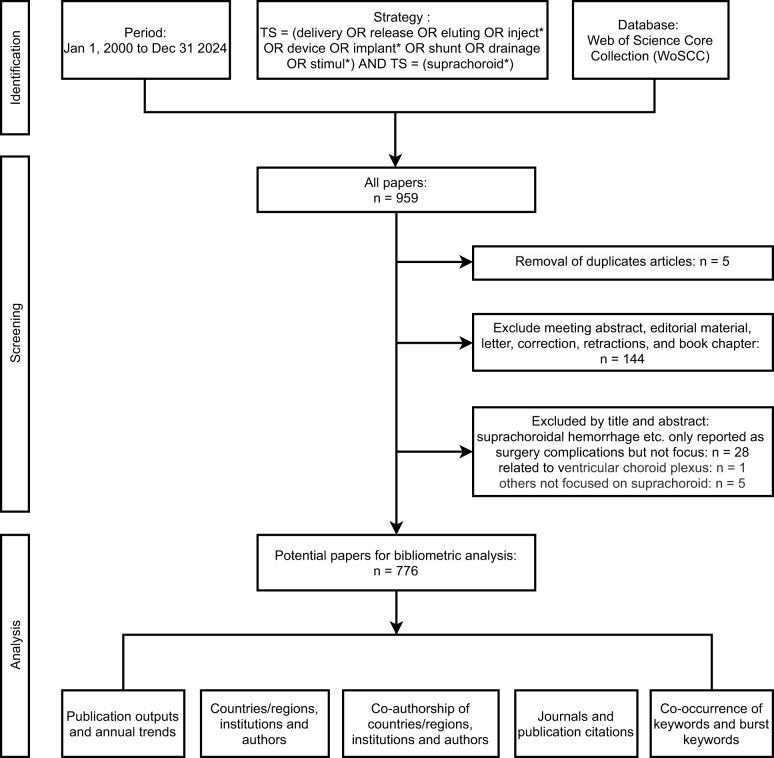
The data collection and retrieval strategy.

In this cross-sectional study, the search for publications to be included was carried out on a single day (15 April 2025). WoSCC was chosen for its high-quality, standardized bibliographic and citation data, and compatibility with bibliometric tools. To avoid analytical bias and ensure data integrity and methodological consistency, WoSCC was selected as the sole data source. The process of data collection and retrieval was shown in Figure 1. The retrieved publications should meet the criteria as followings:

1. The search strategy was “TS = (delivery OR release OR eluting OR inject* OR device OR implant* OR shunt OR drainage OR stimul*) AND TS = (suprachoroid*)”. The search terms were pilot tested and refined to ensure both sensitivity and specificity in capturing relevant publications. Known key studies were used to validate the search strategy, and adjustments were made to ensure comprehensive and accurate retrieval.
2. The publication time was between 2000 and 2024.
3. The publications whose document type were meeting abstract, editorial material, retraction, retracted publication, letter or book chapter were excluded.
4. Only articles published in English were included in the analysis.
5. The following information was retrieved from each publication: authors, article title, source title, keywords, citations, countries/regions, institutions, publication year, WOS categories, and journal impact factors.

### Data analysis and visualization

R package bibliometrix (v4.1.2) was used to quantify the number of publications, journals, and local citations. VOSviewer software (version 1.6.20) was used to create visualized network maps by extracting bibliographic information on authors, institutions, citations, and keywords from WOS downloaded files. Country cooperation network was visualized with the R package circlize (v0.4.15). CiteSpace (v6.3.1) was applied to retrieve citation bursts of keywords. The Journal impact factor (IF) was obtained from the Journal Citation Report 2024.

### Research ethics

All data were obtained and downloaded from WosCC, a publicly available database. The study did not interact with human subjects or animals. Thus, there were no ethical issues and no approval from an Ethics Committee was required.

## Results

### Analysis of publication outputs and annual publication trends

**Figure 2.**
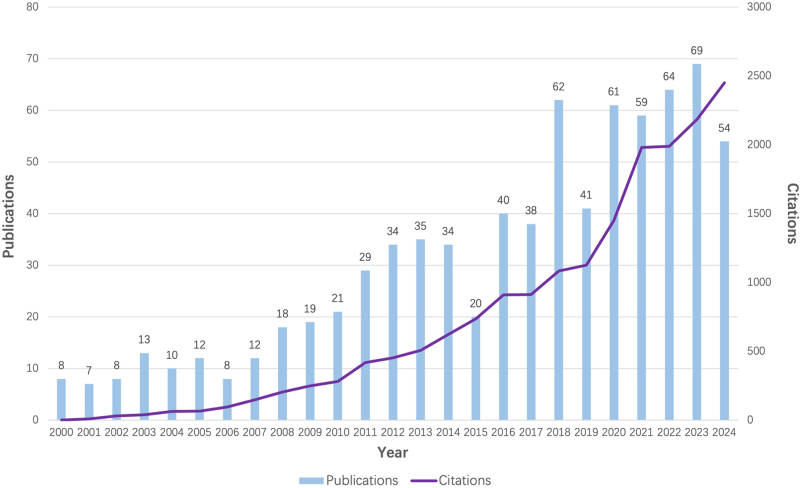
The number of articles and citations on the therapeutic application of SCS per year from 2000 to 2024.

In total, 776 WosCC publications from 2000 to 2024 were retrieved, consisting of 601 (77.6%) research articles and 146 (18.7%) review articles. As is shown in Figure 2, the number of publications steadily increased from 2000 to 2024, with a notable surge beginning in 2018, but had two sharp decreases in 2015 and 2019, respectively. The annual global publications grew from 8 in 2000 to 54 in 2024, representing a 575% increase. The annual publication count peaked at 69 in 2023 (8.9%). Citations also demonstrated a consistent upward trend, particularly after 2020, reaching over 2,500 in 2024. This indicates growing academic interest and impact in the field over the past two decades.

### Distribution of countries/regions, institutions and authors

A total of 59 countries/regions contributed to the publications in the current study. The top 10 most productive countries in terms of the publication numbers are listed in Table 1. The USA accounted for the largest amount of publications (n = 274, 35.3%) with 8343 citations, followed by Australia with 85 publications and Germany with 83 publications. Among Asian countries, Japan, China, India, and South Korea ranked fourth, fifth, seventh, and eighth, respectively.

**Table 1:** The top 10 productive countries/regions

Rank	Country/region	Publications (percentage)	Citations
1	USA	274 (35.3)	8343
2	Australia	85 (11.0)	2607
3	Germany	83 (10.7)	1284
4	Japan	67 (8.6)	1328
5	China	57 (7.3)	552
6	England	38 (4.9)	878
7	India	37 (4.8)	342
8	South Korea	34 (4.4)	966
9	Switzerland	34 (4.4)	998
10	Canada	31 (4.0)	829

These articles were written by 2800 authors from 997 institutions. Table 2 lists the top 10 productive institutions. The first three were University of Melbourne (Australia), Bionics Institute (Australia), and Osaka University (Japan), with 48, 45, and 37 publications, respectively. These institutions represent the most contribution and high level of research in the area of suprachoroidal research field.

**Table 2:** The top 10 productive institutions

Rank	Institution	Country	Total publications (percentage)	Total citations
1	University of Melbourne	Australia	48 (6.2)	1613
2	Bionics Institute	Australia	45 (5.8)	1483
3	Osaka University	Japan	37 (4.8)	1092
4	Nidek Co Ltd	Japan	35 (4.5)	881
5	University of New South Wales Sydney	Australia	32 (4.1)	743
6	Royal Victorian Eye Ear Hospital	Australia	29 (3.7)	959
7	University of California System	USA	28 (3.6)	834
8	Centre for Eye Research Australia	Australia	27 (3.5)	919
9	Emory University	USA	27 (3.5)	1145
10	Nara Institute of Science Technology	Japan	26 (3.4)	262

The top 20 productive authors were listed in Table 3. The works of Fujikado Takashi from Osaka University published the most, with 34 publications and a total of 1069 citations (average 31 citations per publication), and these studies mainly focused on suprachoroidal-transretinal stimulation (STS) prosthesis. The three authors with the most citations were Shivdasani Mohit N, Fujikado Takashi, and Prausnitz Mark R R. The works of Prausnitz Mark R R from Georgia Institute of Technology about SCS microneedle injection and drug delivery had the highest average citations, up to 50 per publication.

**Table 3:** The top 20 productive authors

Rank	Author	Country	Total publications	Total citations	Average citations	Total link strength	H-index
1	Fujikado, Takashi	Japan	34	1069	31	250	48
2	Shivdasani, Mohit N.	Australia	32	1147	36	272	24
3	Terasawa, Yasuo	Japan	27	261	10	168	10
4	Ohta, Jun	Japan	26	255	10	149	33
5	Allen, Penelope J.	Australia	24	752	31	285	20
6	Kanda, Hiroyuki	Japan	23	821	36	174	17
8	Lovell, Nigel H.	Australia	23	652	28	119	60
9	Luu, Chi D.	Australia	22	748	34	267	42
10	Nayagam, David A. X.	Australia	21	741	35	245	19
11	Prausnitz, Mark R. R.	USA	21	1050	50	48	94
12	Suaning, Gregg J.	Australia	20	596	30	99	29
13	Ciulla, Thomas A.	USA	19	443	23	108	55
14	Williams, Chris E.	Australia	19	861	45	180	17
15	Sasagawa, Kiyotaka	Japan	18	181	10	114	24
16	Ayton, Lauren N.	Australia	17	716	42	218	31
17	Morimoto, Takeshi	Japan	17	646	38	139	21
19	Shepherd, Robert K.	Australia	17	869	51	168	50
20	Nishida, Kohji	Japan	16	396	25	184	63

### Co-authorship of countries/regions, institutions and authors

**Figure 3.**
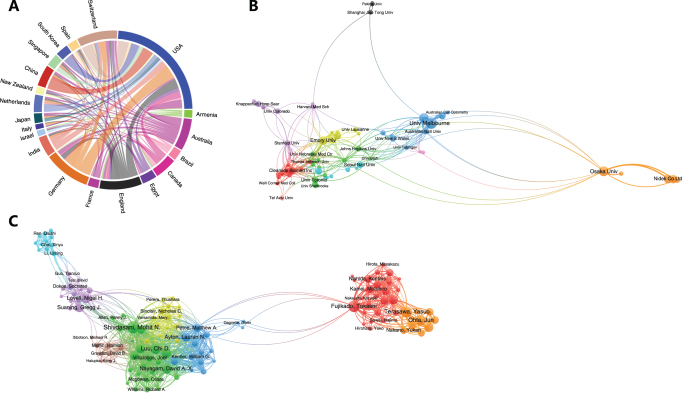
Co-authorship of countries/regions (A), institutions (B), and authors (C). (A) The USA (blue) had the most partners and the highest total link strength. (B) Ten clusters were identified. Centers included the Clearside Biomed Inc. (red), University of Melbourne (blue), and Emory University (yellow). (C) Eight clusters were identified. Fujikado Takashi, Shivdasani Mohit N, Luu Chi D, and Ayton Lauren N expert in suprachoroidal retinal prosthesis were at the centers.

Co-authorship analysis of countries/regions revealed the activity of a country/region in a global collaboration relationship (Fig. 3A). The USA had the most cooperating partners (n = 35) and was closely linked with Germany, China, and Switzerland. Also, the USA had the highest total link strength (n = 142), followed by England (n = 50) and Germany (n = 50). On the other hand, Egypt, Armenia, Brazil, New Zealand, Italy, and France had relatively less cooperation with the USA than with other countries.

We set the minimum number of articles published by the institutions as 5, and VOSviewer was employed for visualization of the co-authorship network of these institutions (Fig. 3B). The co-authorship network, including 75 institutions, was divided into 10 clusters represented by different colors. The largest cluster (in red) consisting of 14 institutions was centered on the Clearside Biomed Inc. The second (in green) included 12 universities from North America, such as Johns Hopkins University, University of Michigan, and University of Toronto, and had no obvious center. Emory University and the University of Michigan had the most significant number of cooperating partners (n = 23), followed by Stanford University (n = 22). As for total link strength, the University of Melbourne (n = 128) and Bionics Institute (n = 107) from Australia were the top two.

When setting minimum articles published by the author as 2, minimum citations as 10, and minimum total link strength as 2, the co-authorship network consists of 119 authors and was divided into eight clusters represented by different colors (Fig. 3C). The largest cluster (in red) was centered on Fujikado Takashi from Japan. Among all the 2800 authors, Shivdasani Mohit N from the University of New South Wales had the most significant number of cooperating partners (n = 98), followed by Luu Chi D (n = 92) and Allen Penelope J (n = 87) from the University of Melbourne. These three authors were in the second largest cluster (in green), and their research focused mainly on suprachoroidal retinal prosthesis. Regarding total link strength (Table 3), they were still the three most important authors in the collaborative research network.

### Analysis of source journals and publication citations

There were 248 journals involved in the publications related to the suprachoroidal research field. As is shown in Table 4, the top 10 journals in terms of the numbers of published papers are listed, accounting for 31.2%. *Investigative Ophthalmology & Visual Science* (IF 5.0) was the most productive journal (50 publications) and the most highly cited journal (2249 citations), followed by *Journal of Glaucoma* (IF 2.0) with 39 publications and *Journal of Neural Engineering* (IF 3.7) with 28 publications.

**Table 4:** The top 10 journals published most on research on suprachoroidal application

Rank	Source	Category	IF (2024)	Number of articles (percentage)	Total citations
1	*Investigative Ophthalmology & Visual Science*	Ophthalmology	4.7	50 (6.4)	2249
2	*Journal of Glaucoma*	Ophthalmology	1.8	39 (5.0)	570
3	*Journal of Neural Engineering*	Engineering, Biomedical	3.8	28 (3.6)	846
4	*Graefes Archive for Clinical and Experimental Ophthalmology*	Ophthalmology	2.3	26 (3.4)	560
5	*Translational Vision Science & Technology*	Ophthalmology	2.6	21 (2.7)	243
6	*Retina-The Journal of Retinal and Vitreous Diseases*	Ophthalmology	2.1	19 (2.5)	298
7	*British Journal of Ophthalmology*	Ophthalmology	3.5	17 (2.2)	696
8	*Ophthalmology*	Ophthalmology	9.5	16 (2.1)	1002
9	*Journal of Cataract and Refractive Surgery*	Ophthalmology	3.2	14 (1.8)	241
10	*Current Opinion in Ophthalmology*	Ophthalmology	2.6	12 (1.5)	779

A total of 776 articles were cited 17910 times, with an average number of 23 citations per document. Table 5 lists the top 11 most locally cited publications that represented fundamental research in our collected studies. Of the top 11 papers, 8 explored the utility of SCS for drug delivery^[15–22]^, and 3 papers described themes related to suprachoroidal retinal prosthesis^[23–25]^. “Targeted Administration into the Suprachoroidal Space Using a Microneedle for Drug Delivery to the Posterior Segment of the Eye,” which was published in *Investigative Ophthalmology & Visual Science* in 2012 was the most locally cited one and ranked the tenth in total citations. “Suprachoroidal Drug Delivery to the Back of the Eye Using Hollow Microneedles” and “First-in-Human Trial of a Novel Suprachoroidal Retinal Prosthesis” were also in the top 10 of total citations. Notably, several first authors (e.g., Olsen TW, Gilger BC, Fujikado T, Yeh S, Ayton LN) had high h-index values (>30), indicating sustained academic impact.

**Table 5:** The top 11 highest locally cited articles

Rank	Title (author)	Year	Journal	Local citations	Total citations (WoSCC)	H-index (first author)
1	Targeted Administration into the Suprachoroidal Space Using a Microneedle for Drug Delivery to the Posterior Segment of the Eye (Patel SR *et al*)	2012	*Investigative Ophthalmology & Visual Science*	86	179	9
2	Suprachoroidal Drug Delivery to the Back of the Eye Using Hollow Microneedles (Patel SR *et al*)	2011	*Pharmaceutical Research*	78	218	9
3	Cannulation of the suprachoroidal space: A novel drug delivery methodology to the posterior segment (Olsen TW *et al*)	2006	*American Journal of Ophthalmology*	76	122	44
4	Treatment of Acute Posterior Uveitis in a Porcine Model by Injection of Triamcinolone Acetonide into the Suprachoroidal Space Using Microneedles (Gilger BC *et al*)	2013	*Investigative Ophthalmology & Visual Science*	67	117	35
5	Testing of Semichronically Implanted Retinal Prosthesis by Suprachoroidal-Transretinal Stimulation in Patients with Retinitis Pigmentosa (Fujikado T *et al*)	2011	*Investigative Ophthalmology & Visual Science*	66	166	48
6	Efficacy and Safety of Suprachoroidal CLS-TA for Macular Edema Secondary to Noninfectious Uveitis Phase 3 Randomized Trial (Yeh S *et al*)	2020	*Ophthalmology*	59	108	38
7	First-in-Human Trial of a Novel Suprachoroidal Retinal Prosthesis (Ayton LN *et al*)	2014	*Plos One*	58	239	31
8	Electrophysiological studies of the feasibility of suprachoroidal-transretinal stimulation for artificial vision in normal and RCS rats (Kanda H *et al*)	2004	*Investigative Ophthalmology & Visual Science*	58	113	17
9	Safety and pharmacodynamics of suprachoroidal injection of triamcinolone acetonide as a controlled ocular drug release model (Chen M *et al*)	2015	*Journal of Controlled Release*	57	80	6
10	Pharmacokinetics of Pars Plana Intravitreal Injections versus Microcannula Suprachoroidal Injections of Bevacizumab in a Porcine Model (Olsen TW *et al*)	2011	*Investigative Ophthalmology & Visual Science*	47	80	44
10	Evaluation of a novel biomaterial in the suprachoroidal space of the rabbit eye (Einmahl S *et al*)	2002	*Investigative Ophthalmology & Visual Science*	47	83	8

### The co-occurrence of keywords and burst keyword detection

**Figure 4.**
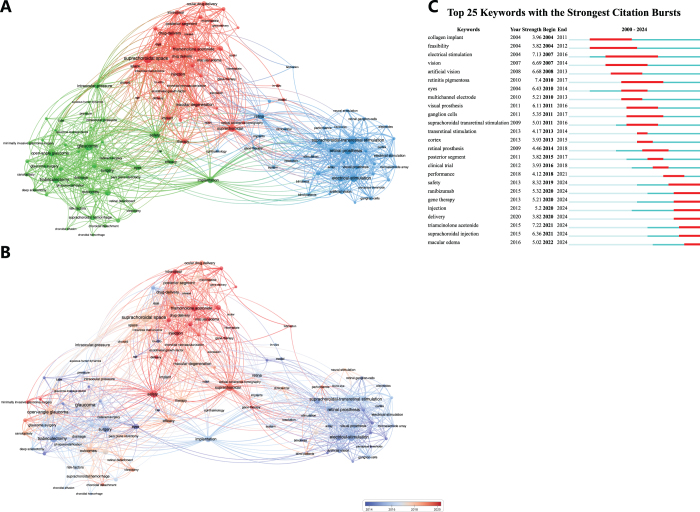
The co-occurrence of keywords (A, B) and burst keyword detection (C). (A) Three clusters were identified. Red cluster was centered on drug-delivery. Green cluster was centered on glaucoma. Blue cluster was centered on suprachoroidal-transretinal stimulation (STS). (B) “Drug delivery” cluster was the hotspot in recent years. (C) Emerging research frontiers included ranibizumab, gene therapy, suprachoroidal injection, triamcinolone acetonide, and macular edema.

A total of 2924 keywords appeared in these 776 publications. Keywords analysis identified the most frequently appeared words and their linkage by VOSviewer. We extracted the keywords with the top 130 occurrences and used VOSviewer to establish three main clusters as shown in Figure 4A.

The main keywords in cluster 1 (in red) included “suprachoroidal space,” “drug-delivery,” “triamcinolone acetonide,” “injection, “microneedle,” “uveitis,” “macular degeneration,” and “gene therapy.” Cluster 2 (in green) was centered on “glaucoma” and other featured keywords included “minimally invasive glaucoma surgery (MIGS),” “trabeculectomy,” “uveoscleral outflow,” “drainage,” and “collagen implant.” The main keywords in cluster 3 (in blue) were “suprachoroidal-transretinal stimulation (STS),” “retinal prosthesis,” and “retinitis-pigmentosa.”

To explore the evolution trends over time, overlay map was created and shown in Figure 4B. Though “glaucoma” cluster and “STS” cluster were studied at a relative early stage, there were still some hot topics such as “MIGS,” “suprachoroidal drainage,” “neural stimulation,” “clinical trial,” “gene therapy,” and “fabrication” in these two clusters. The areas related to “drug delivery” cluster have thrived in recent years, while “pharmakinetics” and “in-vitro” studies were relatively outdated in this cluster.

Figure 4C shows the top 25 burst keywords in this field from 2004 to 2024, representing topics that emerged sharply within a short duration. “Safety” had the highest burst strength (8.32, from 2019), followed by “retinitis pigmentosa” (7.4, from 2010 to 2017) and “triamcinolone acetonide” (7.22, from 2021). Frontier research hotspots recently included “ranibizumab,” “gene therapy,” “(suprachoroidal) injection,” “delivery,” “triamcinolone acetonide,” and “macular edema.”

### Analysis of high-impact publications

To further distinguish high-quality studies, we performed a stratified analysis of the top 10% most cited articles (n = 78) from our dataset (Supplementary Digital Content Table 1, available at: http://links.lww.com/JS9/F22). These publications had citation counts ranging from 62 to 485 (in WoSCC), with a mean of 115 citations. They were published in high-impact journals such as *Advanced Drug Delivery Reviews* (IF: 17.6), *Progress in Retinal and Eye Research* (IF: 14.7), *Journal of Controlled Release* (IF: 11.5), *Ophthalmology* (IF: 9.5), and *Investigative Ophthalmology & Visual Science* (IF: 4.7). Among the top-cited papers, nearly 50% focused on suprachoroidal drug delivery, particularly involving microneedle technology and corticosteroid administration.

**Figure 5.**
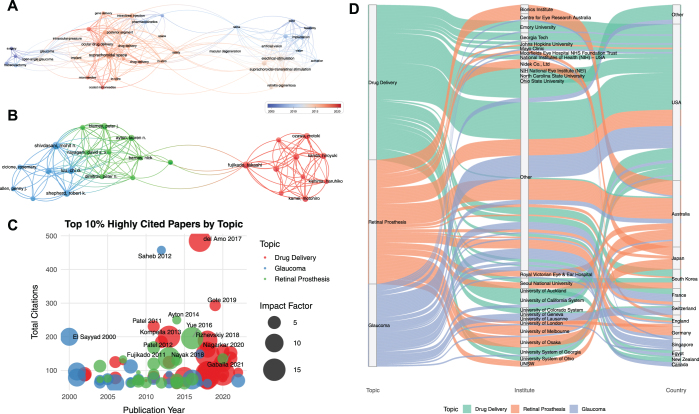
Analysis of the top 10% highly cited publications. (A) Keyword co-occurrence evolution network. The drug delivery cluster has experienced rapid growth in recent years. (B) Co-authorship network. Three prominent groups were detected, including researchers from Japan and Australia, with strong intra-group connectivity. (C) Bubble plot of highly cited papers by topic. Color distinguishes three research areas (red – drug delivery; blue – glaucoma; green – retinal prosthesis). Each bubble represents a single publication, plotted by its publication year (x-axis) and total citations (y-axis). The bubble size corresponds to the impact factor of the source journal. (D) Alluvial diagram showing topic-institute-country relationships. Each stream represents the flow of publications from a topic (left: green – drug delivery; orange – retinal prosthesis; blue – glaucoma) through contributing institutions (middle) to their affiliated countries (right). Top 25 institutions are labeled. The diagram highlights the global collaboration landscape and topic-specific institutional strengths.

Keyword clustering and overlay visualization analyses of highly cited publications revealed topic evolution trends similar to those observed in the full dataset. As shown in Figure 5A, research on glaucoma and retinal prosthesis emerged relatively early, while the drug delivery cluster has experienced rapid growth in recent years. The author collaboration network (Fig. 5B) revealed three distinct clusters, all of which are primarily focused on retinal prosthesis, indicating strong collaborative communities within this subfield. The bubble plot in Figure 5C further supports the topic evolution trends observed in Figure 5A. Publications related to drug delivery tend to appear in journals with higher impact factors and receive more citations. The three most highly cited papers – *del Amo 2017, Saheb 2012*, and *Gote 2019* – are all review articles. Most of the top-cited drug delivery papers were published after 2010, whereas retinal prosthesis publications were more evenly distributed over the past two decades. The alluvial diagram in Figure 5D illustrates the distribution of high-impact publications across research topics, institutions, and countries. Emory University and the University of California System were identified as major contributors to high-impact research in drug delivery, while the University of Melbourne and University of Osaka played leading roles in retinal prosthesis research. Correspondingly, high-impact drug delivery studies were predominantly affiliated in the United States, whereas retinal prosthesis studies were largely concentrated in Australia. In contrast, high-impact publications on glaucoma were more evenly distributed across countries and institutions.

## Discussion

### Global trends in suprachoroidal research

The potential therapeutic application of the suprachoroidal space (SCS) was first described in 1952 by ophthalmologist Smith, R., who reported suprachoroidal air injection for the treatment of retinal detachment[26]. Over the following decades, research on the SCS primarily focused on its role in the uveoscleral outflow pathways of aqueous humor^[27,28]^ and its use in experimental modeling, including the establishment of toxoplasmic retinochoroiditis[29], choroidal melanoma[30], and choroidal neovascularization models[31]. Sporadic reports also explored therapeutic applications such as extraocular muscle implantation[32], suprachoroidal injection of hyaluronic acid for retinal detachment[33], and surgical opening of the SCS in glaucoma^[34,35]^. The modern exploration of the SCS as a clinical treatment route began in the early 21st century, driven by advancements in imaging techniques, biomaterials, microcannulation, and microneedle technology^[16,17,21]^. Several milestone studies emerged during this period. In 2003, the first implantation of an SCS drainage device for glaucoma was reported and highlighted viscoelastic substances to separate the choroid from the sclera[36]. In 2004, the first report of STS introduced a novel method for artificial vision, utilizing suprachoroidal electrode implantation to minimize retinal damage[25]. In 2006, a pivotal development was the introduction of triamcinolone acetonide by suprachoroidal cannulation[17]. Over the past two decades, research has increasingly focused on the use of the SCS for treating retinal and choroidal diseases, including age-related macular degeneration (AMD), diabetic macular edema (DME), and uveitis. These researches, later commercialized as XIPERE® (Clearside Biomedical), demonstrated efficacy and safety in clinical trials, as reported by Yeh *et al*[19]. More recently, large-scale clinical trials have expanded the scope of SCS applications, including gene therapy, viral vector delivery, and the implantation of retinal prosthetics. These advancements underscore the growing recognition of the SCS as a versatile and minimally invasive therapeutic route.

The interdisciplinary nature of the treatment application suprachoroidal research is evident from the distribution of publications across diverse journals, indicating the convergence of clinical, pharmaceutical, and biomedical engineering expertise in advancing this field. The global interest in suprachoroidal research is further underscored by contributions from over 2800 authors affiliated with 997 research institutions across 59 countries. As shown in Figure 3, international collaboration has been a defining feature of this field. The USA emerged as the leading contributor, producing the highest number of publications and actively fostering international research collaboration.

However, among the top 10 most productive institutions in suprachoroidal research, only two were from the USA. This apparent discrepancy can be attributed to the large number of research institutions in the USA, which has resulted in a more dispersed distribution of research output. This phenomenon is also evident in the institutional co-occurrence network shown in Figure 3B, where American institutions are widely distributed across multiple clusters, indicating a decentralized pattern of collaboration. In contrast, Japanese institutions presented a more concentrated research effort. On the other hand, the red cluster and blue cluster in Figure 3B presented a close intra-country collaboration in USA and Australia, respectively.

As shown in Table 3, Fujikado Takashi was identified as the most productive author with a relatively more localized research network. On the other hand, Shivdasani Mohit N. stood out as the author with the highest total citations and the most extensive collaborative network. Both of them are accomplished in the field of Engineering and Neurosciences.

Moreover, our stratified analysis of the top 10% most cited publications revealed clear thematic trends. Most of these studies focused on microneedle-based drug delivery and corticosteroid therapy, which aligns with the recent burst keywords and clinical translation milestones such as the FDA approval of Xipere®. This convergence between bibliometric impact and translational relevance underscores the field’s maturation and its potential for future therapeutic breakthroughs.

### Cluster 1: drug-delivery

Despite the fact that “pharmacokinetics” is a relatively old topic in this cluster, ongoing research endeavors enhance drug utilization in the SCS. The aqueous soluble drugs exhibit a significantly faster clearance rate with SCS delivery than intravitreal (IVT) injections, potentially requiring more frequent injections by SCS^[15,20,22]^. Studies have suggested that changing the viscosity and particle size of materials could slow the clearance^[37,38]^. Also, controlled-release drug delivery systems made of biopolymers such as peptide hydrogel, poly(ortho ester)s, and polyurethane are found to be effective strategies to extend the lifetime of drugs, which has been demonstrated in the sustained release of bevacizumab, dexamethasone, cyclosporine and acriflavine[39]. On the other hand, given the circumferential spread of drugs within the SCS, several strategies are being studied to enhance their targeting and bioavailability at the desired sites. Iontophoresis, swollen hydrogel pushing, high-density particle emulsions, micro-stents, and formulations containing collagenase are under investigation to achieve this goal^[3,37]^.

Our analysis shows the burst of keywords like “ranibizumab,” “gene therapy,” “injection,” “triamcinolone acetonide,” and “macular edema” in recent years, which aligns with clinical interest in using the SCS as a minimally invasive route for posterior segment drug delivery. As shown in Table 6, substantial clinical trials have shown promising results, with the SCS injection of TA (CLS-TA; Clearside Biomedical, Alpharetta, GA, USA) demonstrating both safety and efficacy in treating non-infectious uveitis (NIU), NIU-associated macular edema (NIU-ME), and diabetic macular edema (DME). Based on these results, Xipere® (triamcinolone acetonide injectable suspension) for suprachoroidal use received FDA approval in 2021 for the treatment of UME and is administrated via the specific SCS Microinjector® (Clearside Biomedical, Inc.), which marked a significant milestone in the SCS therapeutic field.

**Table 6:** SCS corticosteroid pharmacotherapies in phase 2 or 3 clinical trials

Disease	Clinical trial	Study phase	Study focus	Group (number of eyes)	Primary outcome measures	Conclusions	Ref.
DME	OXEYE (NCT05697809)	Phase 2	Suprachoroidal sustained-release OXU-001 (dexamethasone microspheres; DEXAspheres®) using the Oxulumis® illuminated microcatheterization device compared with IVT dexamethasone implant (OZURDEX®) for DME	Part A: Mid-dose suprachoroidal OXU-001 (n = 9); High-dose (n = 9)	Adverse events	/ (Active, not recruiting)	[44]
				Part B: suprachoroidal OXU-001 Dose 1 (n = 44); Dose 2 (n = 44); IVT Ozurdex® (n = 22)			
	CAPE (NCT05512962)	Phase 2	Suprachoroidal TA (Triesence®) administered with the Oxulumis® (microcatheterization device for DME)	2.4 mg Triesence® (n = 13); 4 mg Triesence® (n = 12)	Adverse events	Most common adverse event was conjunctival hemorrhage. No difference in AE between two arms. No SAE was observed. High-dose Triesence® group (4 mg) had greater visual benefit.	[45]
	TYBEE (NCT03126786)	Phase 2	Suprachoroidal CLS-TA plus IVT aflibercept versus aflibercept alone for DME	CLS-TA and aflibercept at baseline and week 12 (n = 36); Aflibercept at baseline, week 4, week 8 and week 12 (n = 35)	Mean change from baseline in BCVA	The visual benefit was similar between two arms. Suprachoroidal CLS-TA may reduce treatment burden. Suprachoroidal CLS-TA is safe; no difference in AE between the arms.	[46]
	HULK (NCT02949024)	Phase 1/2	Suprachoroidal CLS-TA alone or in combination with IVT aflibercept for DME	Treatment-naïve group: IVT aflibercept and CLS-TA (n = 10); Previously treated group: CLS-TA monotherapy (n = 10)	Adverse events	Anatomic improvements in all eyes after CLS-TA injection. Suprachoroidal injection of CLS-TA was safe in DME patients. Greater benefit for treatment-naïve eyes with CLS-TA.	[47]
RVO-ME	TANZANITE (NCT02303184)	Phase 2	Suprachoroidal CLS-TA in combination with IVT aflibercept for RVO-ME	CLS-TA plus aflibercept (n = 23); Aflibercept alone (n = 23)	Number of times a subject qualifies to be administered IVT aflibercept	Combination therapy was well tolerated and reduced additional IVT aflibercept injections.	[48]
	SAPPHIRE (NCT02980874)	Phase 3	Suprachoroidal injection of TA with IVT aflibercept for RVO-ME	CLS-TA plus aflibercept (n = 231); Aflibercept alone (n = 229)	Proportion of subjects demonstrating ≥ 15 letter improvement from baseline in ETDRS	No additional benefit of the combination therapy and thus terminated. Good safety of the procedure.	[49]
	TOPAZ (NCT03203447)	Phase 3	Suprachoroidal injection of TA with IVT anti-VEGF for RVO-ME	Lucentis or Avastin plus CLS-TA (n = 162); Lucentis or Avastin alone (n = 163)	Proportion of subjects demonstrating ≥ 15 letter improvement from baseline in ETDRS	/ (Terminated due to the SAPPHIRE trial results.)	[50]
NIU-ME	PEACHTREE (NCT02595398)	Phase 3	Safety and efficacy of suprachoroidal CLS-TA injection for NIU-ME	CLS-TA at day 0 and week 12 (n = 96); Sham procedure at day 0 and week 12 (n = 64)	Number of subjects demonstrating ≥ 15 letter improvement from baseline in BCVA at 24 weeks	Clinically meaningful improvements in vision for nearly 50% of patients.	[51]
	MAGNOLIA (NCT02952001)	Observational	Extension study of the safety and efficacy of CLS-TA for treatment of NIU-ME	CLS-TA at day 0 and week 12 (n = 28); Sham procedure at day 0 and week 12 (n = 5)	Number of subjects demonstrating ≥ 15 letter improvement from baseline in BCVA at 24 weeks	Approximately 50% of patients did not require additional treatment for up to 9 months following the last CLS-TA administration.	[52]
NIU	AZALEA (NCT03097315)	Phase 3	Open-label safety trial for suprachoroidal CLS-TA for NIU	Two suprachoroidal injections of CLS-TA at baseline and week 12 (n = 38)	Adverse events	Suprachoroidal injection of CLS-TA was safe and well tolerated. Efficacy parameters showed improvement over 24 weeks (signs of inflammation and the need of rescue therapy).	[53]

DME: diabetic macular edema; BCVA: best corrected visual acuity; AE: adverse events; ETDRS: early treatment of diabetic retinopathy study; IVT: intravitreal; RVO-ME: macular edema following retinal vein occlusion; NIU-ME: macular edema secondary to noninfectious uveitis.

In the field of ophthalmic oncology, AU-011 (belzupacap sarotalocan), a virus-like drug conjugate is currently in clinical investigation for the treatment of small choroidal melanoma. In addition, the suprachoroidal autograft by Limoli Retinal Restoration Technique (LLRT) indicated efficacy in dry AMD^[40,41]^. retinitis pigmentosa and glaucomatous optic neuropathy patients^[42,43]^. The implanted cells included adipocytes, adipose-derived stem cells, and platelets, leading to the secretion of growth factors in the choroidal area to achieve retinal neuroenhancement.

The success of the suprachoroidal drug delivery described above has not been demonstrated in macular edema following retinal vein occlusion (RVO-ME) though. The failure of the SAPPHIRE trial underscores the difficulty of demonstrating incremental benefits of combination therapies in diseases like RVO-ME, where highly effective monotherapies already exist. In addition, the treatment burden reduction did not translate into clinically meaningful functional gains, and was insufficient to justify the added complexity, cost, and potential side effects of combination therapy. Future studies may need to focus on specific subpopulations who might benefit from combination therapy or exploring alternative endpoints.

While “gene therapy” is an emerging research hotspot, the clinical translation of this approach remains in its early stages and still a largely experimental approach. Currently, only a few clinical trials have been registered to investigate suprachoroidal gene therapy. For example, RGX-314, an AAV8-delivered anti-VEGF gene therapy is being evaluated in patients with nAMD (NCT04514653) and diabetic retinopathy (NCT04567550). All of these trials are still in the recruitment phase, and no clinical outcomes have been published as of July 2025. In contrast, most published evidence on SCS gene therapy is currently limited to preclinical studies, including investigations into AAV vector delivery efficiency, immune responses, and novel non-viral delivery methods in animal models^[54–58]^.

**Figure 6.**
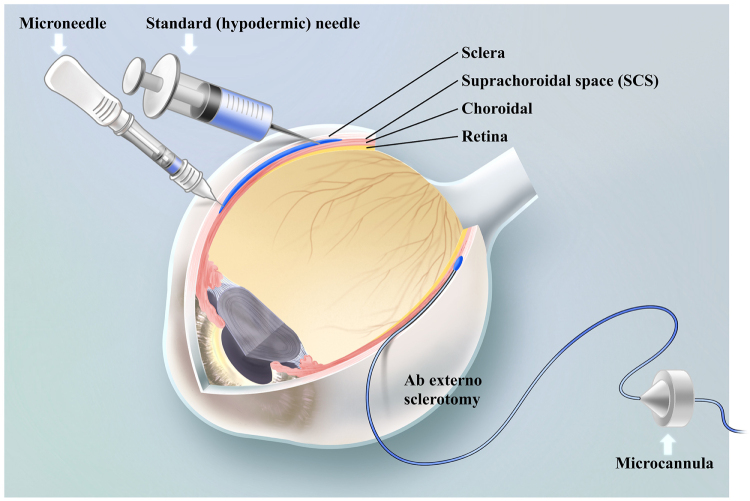
Illustration of three devices/techniques for suprachoroidal drug delivery. Microcannula-guided ab externo sclerotomy allows precise SCS targeting. Standard hypodermic needles rely on tactile feedback for scleral penetration. Hollow microneedles tailored to scleral thickness enable safe and efficient suprachoroidal access.

The advent of technological innovations, especially the invention of hollow microneedles, has precipitated a surge in the field of SCS drug delivery (Fig. 6). Traditional surgical approaches involve ab externo sclerotomy to access the SCS, allowing precise targeting with catheter-guided visualization^[17,20,59]^. However, these methods are invasive, require operating room settings, and carry risks such as choroidal hemorrhage, retinal detachment, and postoperative inflammation[17]. Standard hypodermic needles offer a less invasive alternative but lack visualization, relying on tactile feedback for scleral penetration[22]. This approach requires significant expertise and is associated with risks such as inadvertent intravitreal injection and choroidal hemorrhage[60]. Hollow microneedles, specifically designed for suprachoroidal delivery, are engineered to match the scleral thickness, preventing deeper penetration^[15,61]^. They allow circumferential drug distribution within the SCS, can be performed in outpatient settings with minimal training, and have demonstrated consistent safety and efficacy in abundant clinical trial procedures[62]. More recently, an advanced microneedle injector incorporating resistance-sensing technology has been introduced[63]. This mechanical system automatically detects the sudden loss of resistance upon entering the SCS, halting needle advancement and initiating drug delivery. By adapting to varying eye sizes and scleral thicknesses, it broadens the applicability of suprachoroidal injections across diverse patient populations. In conclusion, microneedle injection represents a significant advancement in suprachoroidal drug delivery, with its simplicity and favorable safety profile positioning it as the most promising method for clinical use.

### Cluster 2: glaucoma

**Figure 7.**
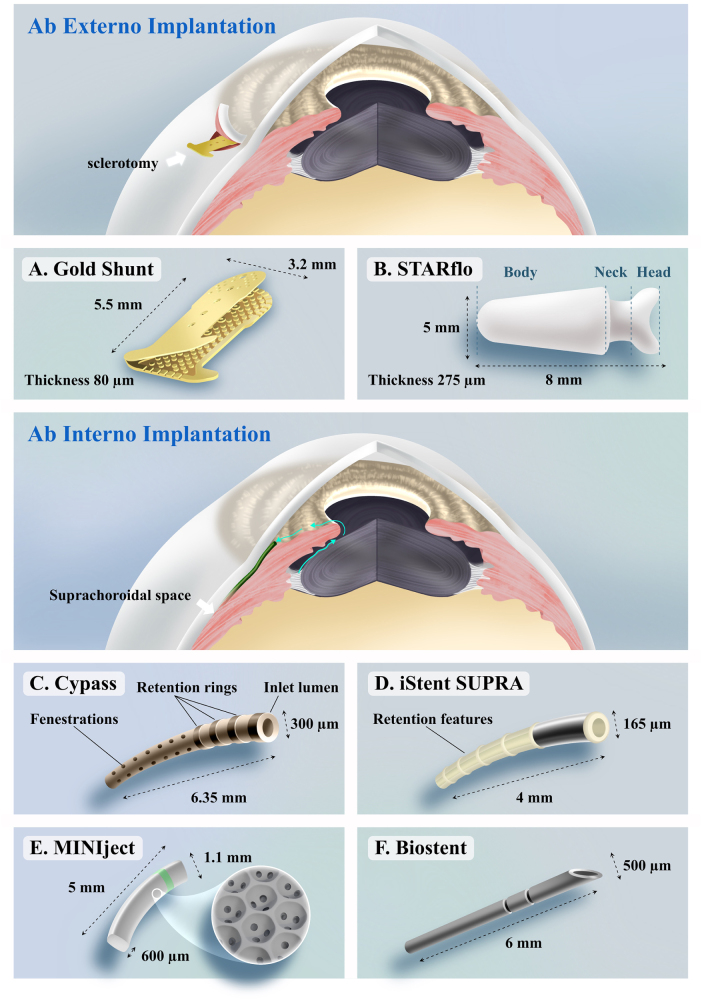
Ab externo (A–B) and ab interno (C–F) suprachoroidal shunts. Ab externo: external surgical approach through the sclera incision into the SCS. Ab interno: device is inserted from inside the eye, typically through the anterior chamber into the SCS.

In this cluster, the transition of keywords over the past two decades from “trabeculectomy” to “MIGS” reflects a shift in glaucoma treatment paradigms toward safer, less invasive procedures. While traditional procedures effectively reduce IOP, they bear the potential for hypotony-induced impairments and associated bleb-related complications[64]. In the pursuit of attaining enhanced safety alongside adequate efficacy, novel surgical modalities have emerged, i.e., suprachoroidal shunts, including ab externo and ab interno approach (Fig. 7). The uveoscleral route is an important aqueous outflow conduit besides the trabecular route, which is driven by the negative pressure gradient between the anterior chamber and the supraciliary space. The optimal suprachoroidal device would ideally provide a consistent and long-lasting reduction in IOP without triggering a significant fibroblastic response that could lead to scarring and treatment failure.

A representative of ab externo suprachoroidal shunts is Gold Shunt (SOLX Inc., Waltham, Massachusetts, USA), which is a double-plated fenestrated device made of 24-karat gold[65] (Fig. 7A). During the operation, a scleral flap is dissected, and then the anterior portion of the shunt is inserted into the anterior chamber of the eye, while the posterior aspect is placed in the SCS. The scleral flap is carefully sutured closed to ensure a watertight seal before the conjunctiva is closed, aiming to prevent the formation of a bleb[66]. The ab externo technique offers certain advantages in cases where corneal disease restricts a clear gonioscopic view required for ab interno surgery. However, the SOLX Gold Shunt has a high failure rate associated with significant fibrotic tissue formation within and around the device. Additionally, severe complications, including retinal detachment, endophthalmitis, and suprachoroidal hemorrhage, have been reported^[67,68]^. The STARflo (iStar Medical, Wavre, Belgium), made of microporous silicone, is another device implanted in the SCS using an ab externo approach (Fig. 7B). However, limited clinical data and a 24-month study indicate inadequate IOP reduction and complications such as corneal decompensation, hypotony, and choroidal hemorrhage, questioning its long-term efficacy[69].

The ab interno approach offers the advantage of avoiding manipulation of the conjunctiva and sclera, allowing for potential future incisional glaucoma surgery if needed. It can also be easily combined with cataract surgery through the same clear corneal incision. CyPass Micro-Stent (Alcon, Fort Worth, TX) is a 6.35 mm-long cylindrical polyimide implant that received US FDA approval in 2016 (Fig. 7C). During the surgery, the guidewire is introduced into the anterior chamber and slightly dissects the ciliary body from the scleral spur[70]. Complications of CyPass include IOP elevations, hypotony, transient hyphema, and progression of cataracts[4]. However, due to the high endothelial cell loss rates based on the results of a 5-year follow-up, the CyPass Micro-Stent was voluntarily withdrawn from the market in 2018^[71,72]^. The iStent SUPRA (Glaukos Corporation in California, USA) is a curve tube made of heparin-coated polyethersulfone with porous titanium coating (Fig. 7D). While the device has shown potential in reducing intraocular pressure, its long-term efficacy and safety still require further investigation and validation[73]. The MINIject (iSTAR Medical, Wavre, Belgium) made of microporous silicone which has outstanding anti-fibrotic properties and biocompatibility (Fig. 7E) also showed promising outcomes[74]. However, similar to the CyPass, adverse events of IOP rise have been reported[75]. Larger clinical trials of MINIject are currently underway. Biostent (IANTREK), a highly permeable, homologous biotissue scleral allograft (Fig. 7F), has also shown safety and efficacy in its first-in-human study[76].

While “MIGS” was an emerging hot topic in our bibliometric analysis, its frequency of occurrences was relatively small (Fig. 4B), and it was not covered by the top 10 highest cited articles (Table 5). Nevertheless, the bibliometric trend suggests sustained research interest and ongoing innovation in this area, particularly in exploring biomaterials and surgical techniques that improve long-term outcomes.

### Cluster 3: suprachoroidal-transretinal stimulation

Although earlier research focused on proof-of-concept and device safety, recent bibliometric indicators point to sustained interest in refining suprachoroidal implants for visual prosthetics. This field exhibits a strong integration of medical and computer science disciplines. Here, we will mainly discuss the clinical aspects of suprachoroidal stimulation.

The bionic eye, also referred to as retinal prosthesis, offers hope for individuals suffering from blinding retinal diseases such as AMD and retinitis pigmentosa, where the loss of photoreceptors leads to vision impairment. Exciting advancements have been made in retinal prosthesis technology in the last decade, leading to the development of various implant types, including epiretinal, subretinal, and suprachoroidal sensors. These implants function by electrically stimulating the remaining cells in the retina, thereby generating visual sensations for the patients.

Suprachoroidal implants offer distinct advantages over other types of implants, as they are considered less technically and surgically challenging, reducing the need for retinal incisions with less complications[24]. Researchers assessed its performance in the real-world setting in patients with late-stage retinitis pigmentosa and found that visual tasks such as washing dishes and identifying doorways showed the greatest benefits over time[77]. However, visual information and quality provided by the device were limited[77]. Still, suprachoroidal microelectrode array implant has a lot to overcome before large-scale clinical application, such as how to provide effective electrical stimulation, high-resolution and selective activation of target cells, and how to customize to every patient[78].

**Figure 8.**
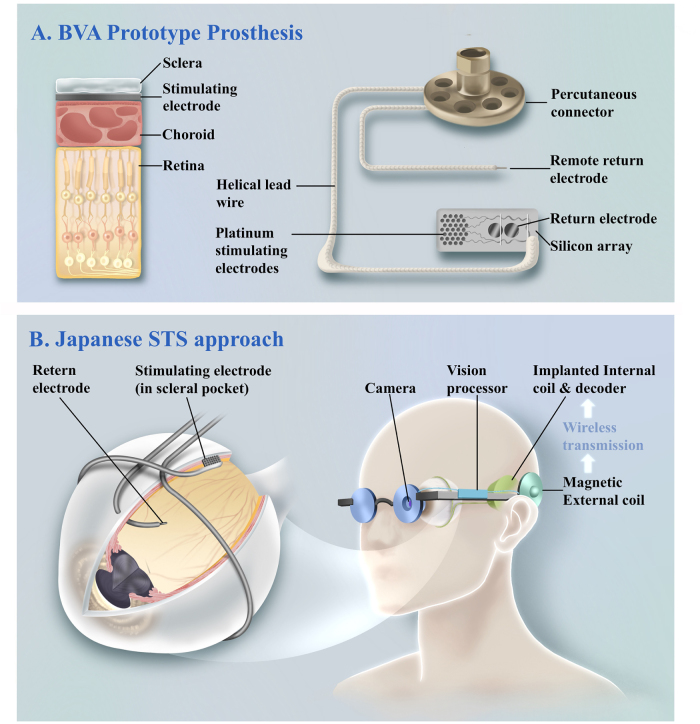
Suprachoroidal retinal prosthesis. (A) Prototype from a first-in-human trial: a 33-electrode silicone array implanted in the SCS, with return electrodes in the SCS and subcutaneously behind the ear, connected via a percutaneous interface for stimulation and monitoring. (B) Fully implantable device with scleral-pocket electrode array and wireless input from a spectacle-mounted CCD camera, enabling out-of-lab use.

Figure 8A describes a percutaneously connected suprachoroidal retinal prosthesis prototype device of the Bionic Vision Australia (BVA) in the first-in-human trial[24]. The electrode array implanted in the SCS was composed of a silicone substrate with 33 platinum electrodes and two large return electrodes. A remote platinum return electrode was implanted under the skin behind the ear. A percutaneous connector allowed flexible neurostimulation and electrode monitoring.

In contrast, the Japanese approach was fully implantable, so the participants could use the device outside of the laboratory environment^[23,79]^. The electrode array was fixed inside a scleral pocket to get a better stability. The return electrode was implanted into the vitreous cavity or subdermal decoder package. A charge-coupled device (CCD) video camera mounted on spectacles captured visual information from the surrounding environment. The acquired image data were then wirelessly transmitted to the internal coil and decoder, delivering corresponding electrical impulses to individual electrodes (Fig. 8B).

Of note, the efficacy of implantable retinal devices for treating retinal diseases is currently under discussion, given the emergence of alternative approaches such as stem cell transplantation and gene therapy, along with optogenetics introducing photosensitive proteins. These developments are reflected in the shifting keyword dynamics, open up new possibilities, and raise questions about the future role of implantable electrode devices.

### Challenges encountered by suprachoroidal research

Despite the promising advancements in suprachoroidal research, several challenges persist. One of the primary challenges lies in the pharmacokinetics of suprachoroidal drug delivery. Though some strategies have shown promise in slowing down the clearance of aqueous-soluble drug in SCS, their translation into consistent clinical outcomes requires further optimization and validation. Additionally, ensuring precise circumferential drug spread and bioavailability at the desired site remains a technical hurdle.

Secondly, in surgical interventions, challenges related to the long-term efficacy and safety of suprachoroidal devices persist. Devices such as the CyPass Micro-Stent initially showed promise but were later withdrawn due to complications like endothelial cell loss.

Furthermore, for suprachoroidal retinal prostheses, suprachoroidal implants struggle to deliver high-resolution visual information and selective activation of retinal cells. Finally, the high cost of suprachoroidal technologies, combined with the need for specialized equipment and expertise, remains a barrier to accessibility.

### Future perspectives

SCS holds significant promise for future therapeutic advancements in ophthalmology. One exciting avenue is its potential for the localized treatment of choroidal melanoma, where precise delivery of chemotherapeutic agents or radiotherapy-enhancing compounds could maximize efficacy while minimizing systemic toxicity. In the management of high myopia, axial elongation could be stabilized by suprachoroidal injections of scleral cross-linking agents[80] and agents increasing choroidal permeability and inhibiting scleral glycosaminoglycan synthesis^[81,82]^. Furthermore, the SCS is being explored for implanting intraocular pressure sensors, which enables continuous monitoring and personalized glaucoma management[83]. The SCS also shows potential for stem cell-based therapies, such as delivering human pluripotent stem cell-derived endothelial cells to repair choroidal ischemia[84].

In the future, precision drug delivery will be a key area of focus. Ongoing developments in carriers, such as nanoparticles, liposomes, and hydrogels, are expected to enhance the bioavailability, stability, and controlled release of therapeutics within the SCS. Meanwhile, the refinement of creating access channels to SCS is another critical area of progress. Innovative devices are expected to overcome the limitations of current hollow microneedles by achieving greater compatibility with diverse drug formulations and adapting to individual anatomical variability.

Recently, the explosive development of artificial intelligence (AI) has opened up new opportunities for this field. For example, AI-driven predictive modeling could simulate drug dispersion and clearance, helping researchers design more effective formulations and delivery systems. Furthermore, AI-powered image analysis could enhance preoperative planning and intraoperative guidance, improving surgical precision. Personalized AI algorithms could also help tailor prosthetic functions to individual patient needs. However, the integration of AI models into clinical workflows necessitates large, high-quality datasets, which are currently limited in this field. Besides, the ethical and regulatory challenges associated with AI, including data privacy, algorithm transparency, and equitable access, add another layer of complexity to its integration into suprachoroidal research.

### Limitations

While bibliometric analysis provides a quantitative and objective overview, it has inherent limitations. First, the data used in this study were exclusively retrieved from the WoSCC database, which may not include all relevant publications from other databases or grey literature. Second, this study did not assess the quality or impact of individual publications, treating all articles equally in the analysis. This potentially skewed the interpretation of key trends and influential contributions. Third, this is not a systematic review or meta-analysis, and thus does not provide quantitative comparisons of therapeutic efficacy or safety among different treatment strategies. Therefore, the findings should be interpreted as reflective of research trends rather than direct evidence for clinical decision-making. Lastly, the reliance on publication and citation metrics may overlook recent advances that have not yet accumulated citations but may be of high clinical significance.

## Conclusions

This study represents the first comprehensive bibliometric analysis of research on the therapeutic application of SCS by providing a visualized overview of research trends, international collaborations, and key focus areas in this field. The findings highlight the steady growth of interest in suprachoroidal research, and reveal emerging themes such as drug delivery systems, surgical interventions, and retinal prosthetics. The analysis of high-impact studies underscores the translational nature of this field, bridging engineering innovations with clinical application. This study serves as a foundation for identifying research priorities and fostering interdisciplinary collaboration in this evolving field.

## Supplementary Material

## Data Availability

The data in this study are publicly available.
