# Supplementary material for: Bibliometric and visualized analysis of the therapeutic application of suprachoroidal space from 2000 to 2024
Source: Int J Surg. 2025 Sep 8;112(1):1635–51. doi: 10.1097/JS9.0000000000003384 (PMC12825562; doi:10.1097/JS9.0000000000003384)
Supplement: Supplementary file 1 [file js9-112-1635-001.docx]

| Publication Year | Times Cited, WoS Core | IF | Topic | Article Title | Source Title | First author |
| --- | --- | --- | --- | --- | --- | --- |
| 2017 | 485 | 14.7 | Drug Delivery | Pharmacokinetic aspects of retinal drug delivery | PROGRESS IN RETINAL AND EYE RESEARCH | del Amo |
| 2012 | 457 | 2.6 | Glaucoma | Micro-invasive glaucoma surgery: current perspectives and future directions | CURRENT OPINION IN OPHTHALMOLOGY | Saheb |
| 2019 | 293 | 3.8 | Drug Delivery | Ocular Drug Delivery: Present Innovations and Future Challenges | JOURNAL OF PHARMACOLOGY AND EXPERIMENTAL THERAPEUTICS | Gote |
| 2014 | 250 | 2.6 | Retinal Prosthesis | First-in-Human Trial of a Novel Suprachoroidal Retinal Prosthesis | PLOS ONE | Ayton |
| 2011 | 231 | 4.3 | Drug Delivery | Suprachoroidal Drug Delivery to the Back of the Eye Using Hollow Microneedles | PHARMACEUTICAL RESEARCH | Patel |
| 2013 | 200 | 14.7 | Drug Delivery | Nanomedicines for back of the eye drug delivery, gene delivery, and imaging | PROGRESS IN RETINAL AND EYE RESEARCH | Kompella |
| 2000 | 200 | 9.5 | Glaucoma | Nonpenetrating deep sclerectomy versus trabeculectomy in bilateral primary open angle glaucoma | OPHTHALMOLOGY | El Sayyad |
| 2016 | 196 | 14.7 | Retinal Prosthesis | Retinal stimulation strategies to restore vision: Fundamentals and systems | PROGRESS IN RETINAL AND EYE RESEARCH | Yue |
| 2018 | 189 | 11.5 | Drug Delivery | Microneedles as the technique of drug delivery enhancement in diverse organs and tissues | JOURNAL OF CONTROLLED RELEASE | Rzhevskiy |
| 2012 | 185 | 4.7 | Drug Delivery | Targeted Administration into the Suprachoroidal Space Using a Microneedle for Drug Delivery to the Posterior Segment of the Eye | INVESTIGATIVE OPHTHALMOLOGY & VISUAL SCIENCE | Patel |
| 2011 | 168 | 4.7 | Retinal Prosthesis | Testing of Semichronically Implanted Retinal Prosthesis by Suprachoroidal-Transretinal Stimulation in Patients with Retinitis Pigmentosa | INVESTIGATIVE OPHTHALMOLOGY & VISUAL SCIENCE | Fujikado |
| 2018 | 162 | 7.5 | Drug Delivery | A review on recent drug delivery systems for posterior segment of eye | BIOMEDICINE & PHARMACOTHERAPY | Nayak |
| 2020 | 158 | 4.9 | Drug Delivery | A review of recent advances in microneedle technology for transdermal drug delivery | JOURNAL OF DRUG DELIVERY SCIENCE AND TECHNOLOGY | Nagarkar |
| 2021 | 152 | 5.5 | Drug Delivery | Corticosteroids in ophthalmology: drug delivery innovations, pharmacology, clinical applications, and future perspectives | DRUG DELIVERY AND TRANSLATIONAL RESEARCH | Gaballa |
| 2017 | 149 | 2.7 | Glaucoma | Unconventional aqueous humor outflow: A review | EXPERIMENTAL EYE RESEARCH | Johnson |
| 2018 | 146 | 17.6 | Drug Delivery | Overcoming ocular drug delivery barriers through the use of physical forces | ADVANCED DRUG DELIVERY REVIEWS | Huang |
| 2021 | 136 | 5.5 | Drug Delivery | Ocular Drug Delivery to the Retina: Current Innovations and Future Perspectives | PHARMACEUTICS | Kim |
| 2013 | 136 | 14.9 | Retinal Prosthesis | Visual prostheses for the blind | TRENDS IN BIOTECHNOLOGY | Shepherd |
| 2020 | 135 | 3.6 | Retinal Prosthesis | An update on retinal prostheses | CLINICAL NEUROPHYSIOLOGY | Ayton |
| 2007 | 133 | 2.3 | Retinal Prosthesis | Evaluation of phosphenes elicited by extraocular stimulation in normals and by suprachoroidal-transretinal stimulation in patients with retinitis pigmentosa | GRAEFES ARCHIVE FOR CLINICAL AND EXPERIMENTAL OPHTHALMOLOGY | Fujikado |
| 2014 | 130 | 3.5 | Retinal Prosthesis | Retinal implants: a systematic review | BRITISH JOURNAL OF OPHTHALMOLOGY | Chuang |
| 2007 | 126 | 1.9 | Drug Delivery | Transport barriers in transscleral drug delivery for retinal diseases | OPHTHALMIC RESEARCH | Kim |
| 2020 | 125 | 5.2 | Drug Delivery | A practical guide to the development of microneedle systems - In clinical trials or on the market | INTERNATIONAL JOURNAL OF PHARMACEUTICS | Lee |
| 2013 | 125 | 4.7 | Drug Delivery | Treatment of Acute Posterior Uveitis in a Porcine Model by Injection of Triamcinolone Acetonide Into the Suprachoroidal Space Using Microneedles | INVESTIGATIVE OPHTHALMOLOGY & VISUAL SCIENCE | Gilger |
| 2006 | 125 | 4.2 | Drug Delivery | Cannulation of the suprachoroidal space: A novel drug delivery methodology to the posterior segment | AMERICAN JOURNAL OF OPHTHALMOLOGY | Olsen |
| 2020 | 122 | 9.5 | Drug Delivery | Efficacy and Safety of Suprachoroidal CLS-TA for Macular Edema Secondary to Noninfectious Uveitis Phase 3 Randomized Trial | OPHTHALMOLOGY | Yeh |
| 2011 | 120 | 4.7 | Retinal Prosthesis | Stimulation with a Wireless Intraocular Epiretinal Implant Elicits Visual Percepts in Blind Humans | INVESTIGATIVE OPHTHALMOLOGY & VISUAL SCIENCE | Klauke |
| 2004 | 113 | 4.7 | Retinal Prosthesis | Electrophysiological studies of the feasibility of suprachoroidal-transretinal stimulation for artificial vision in normal and RCS rats | INVESTIGATIVE OPHTHALMOLOGY & VISUAL SCIENCE | Kanda |
| 2008 | 112 | 5.9 | Glaucoma | Nonpenetrating Glaucoma Surgery | SURVEY OF OPHTHALMOLOGY | Mendrinos |
| 2019 | 110 | 4.3 | Drug Delivery | Recent advances in microneedle-based drug delivery: Special emphasis on its use in paediatric population | EUROPEAN JOURNAL OF PHARMACEUTICS AND BIOPHARMACEUTICS | Duarah |
| 2018 | 99 | 11.5 | Drug Delivery | Ocular drug delivery targeted by iontophoresis in the suprachoroidal space using a microneedle | JOURNAL OF CONTROLLED RELEASE | Jung |
| 2001 | 97 | 9.5 | Glaucoma | Intermediate-term outcomes of 350-mm2 Baerveldt glaucoma implants | OPHTHALMOLOGY | Krishna |
| 2019 | 96 | 3.6 | Drug Delivery | AAV8-vectored suprachoroidal gene transfer produces widespread ocular transgene expression | JOURNAL OF CLINICAL INVESTIGATION | Ding |
| 2022 | 94 | 3 | Drug Delivery | Considerations for Polymers Used in Ocular Drug Delivery | FRONTIERS IN MEDICINE | Allyn |
| 2009 | 94 | 1.4 | Retinal Prosthesis | Focal activation of the feline retina via a suprachoroidal electrode array | VISION RESEARCH | Wong |
| 2020 | 91 | 17.6 | Drug Delivery | Non-transdermal microneedles for advanced drug delivery | ADVANCED DRUG DELIVERY REVIEWS | Lee |
| 2018 | 91 | 17.6 | Drug Delivery | In vitro and ex vivo models to study drug delivery barriers in the posterior segment of the eye | ADVANCED DRUG DELIVERY REVIEWS | Peynshaert |
| 2002 | 89 | 5.9 | Drug Delivery | Drug delivery to the posterior segment from drops | SURVEY OF OPHTHALMOLOGY | Maurice |
| 2020 | 89 | 4.7 | Drug Delivery | Suprachoroidal and Subretinal Injections of AAV Using Transscleral Microneedles for Retinal Gene Delivery in Nonhuman Primates | MOLECULAR THERAPY-METHODS & CLINICAL DEVELOPMENT | Yiu |
| 2013 | 89 | 1.9 | Glaucoma | Update on Minimally Invasive Glaucoma Surgery (MIGS) and New Implants | JOURNAL OF OPHTHALMOLOGY | Brandao |
| 2011 | 89 | 3.8 | Retinal Prosthesis | Electric crosstalk impairs spatial resolution of multi-electrode arrays in retinal implants | JOURNAL OF NEURAL ENGINEERING | Wilke |
| 2002 | 86 | 4.7 | Drug Delivery | Evaluation of a novel biomaterial in the suprachoroidal space of the rabbit eye | INVESTIGATIVE OPHTHALMOLOGY & VISUAL SCIENCE | Einmahl |
| 2018 | 82 | 17.6 | Drug Delivery | The suprachoroidal space as a route of administration to the posterior segment of the eye | ADVANCED DRUG DELIVERY REVIEWS | Chiang |
| 2015 | 81 | 11.5 | Drug Delivery | Safety and pharmacodynamics of suprachoroidal injection of triamcinolone acetonide as a controlled ocular drug release model | JOURNAL OF CONTROLLED RELEASE | Chen |
| 2011 | 80 | 4.7 | Drug Delivery | Pharmacokinetics of Pars Plana Intravitreal Injections versus Microcannula Suprachoroidal Injections of Bevacizumab in a Porcine Model | INVESTIGATIVE OPHTHALMOLOGY & VISUAL SCIENCE | Olsen |
| 2001 | 79 | 9.5 | Glaucoma | Delayed suprachoroidal hemorrhage after glaucoma filtration procedures | OPHTHALMOLOGY | Tuli |
| 2006 | 78 | 4.7 | Drug Delivery | A novel bioerodible deep scleral lamellar cyclosporine implant for uveitis | INVESTIGATIVE OPHTHALMOLOGY & VISUAL SCIENCE | Gilger |
| 2020 | 78 | 2.6 | Drug Delivery | Gene therapy beyond luxturna: a new horizon of the treatment for inherited retinal disease | CURRENT OPINION IN OPHTHALMOLOGY | Prado |
| 2013 | 77 | 4.5 | Drug Delivery | Light-Activated, In Situ Forming Gel for Sustained Suprachoroidal Delivery of Bevacizumab | MOLECULAR PHARMACEUTICS | Tyagi |
| 2011 | 76 | 4.7 | Glaucoma | Miniaturized High-Intensity Focused Ultrasound Device in Patients with Glaucoma: A Clinical Pilot Study | INVESTIGATIVE OPHTHALMOLOGY & VISUAL SCIENCE | Aptel |
| 2005 | 76 | 2.3 | Retinal Prosthesis | Transretinal electrical stimulation by an intrascleral multichannel electrode array in rabbit eyes | GRAEFES ARCHIVE FOR CLINICAL AND EXPERIMENTAL OPHTHALMOLOGY | Nakauchi |
| 2005 | 74 | 4.2 | Glaucoma | Complications following ex-press glaucoma shunt implantation | AMERICAN JOURNAL OF OPHTHALMOLOGY | Stewart |
| 2016 | 74 | 4.7 | Retinal Prosthesis | One-Year Outcome of 49-Channel Suprachoroidal-Transretinal Stimulation Prosthesis in Patients With Advanced Retinitis Pigmentosa | INVESTIGATIVE OPHTHALMOLOGY & VISUAL SCIENCE | Fujikado |
| 2016 | 73 | 11.5 | Glaucoma | Sustained reduction of intraocular pressure by supraciliary delivery of brimonidine-loaded poly(lactic acid) microspheres for the treatment of glaucoma | JOURNAL OF CONTROLLED RELEASE | Chiang |
| 2009 | 73 | 4 | Glaucoma | The Ex-PRESS glaucoma shunt versus trabeculectomy in open-angle glaucoma: a prospective randomized study | ADVANCES IN THERAPY | de Jong |
| 2004 | 73 | 1.9 | Retinal Prosthesis | Transretinal electrical stimulation with a suprachoroidal multichannel electrode in rabbit eyes | JAPANESE JOURNAL OF OPHTHALMOLOGY | Sakaguchi |
| 2004 | 71 | 3.5 | Glaucoma | Comparative study between deep sclerectomy with and without collagen implant: long term follow up | BRITISH JOURNAL OF OPHTHALMOLOGY | Shaarawy |
| 2014 | 71 | 5.2 | Retinal Prosthesis | A review and update on the current status of retinal prostheses (bionic eye) | BRITISH MEDICAL BULLETIN | Luo |
| 2009 | 71 | 3.5 | Retinal Prosthesis | Implantable CMOS Biomedical Devices | SENSORS | Ohta |
| 2018 | 70 | 9.6 | Drug Delivery | Intracorneal injection of a detachable hybrid microneedle for sustained drug delivery | ACTA BIOMATERIALIA | Lee |
| 2010 | 70 | 1.3 | Drug Delivery | Long-term outcome after implantation of a suprachoroidal cyclosporine drug delivery device in horses with recurrent uveitis | VETERINARY OPHTHALMOLOGY | Gilger |
| 2022 | 70 | 5.6 | Glaucoma | The surgical management of glaucoma: A review | CLINICAL AND EXPERIMENTAL OPHTHALMOLOGY | Lim |
| 2008 | 70 | 1.8 | Glaucoma | Ten Years Follow-up After Deep Sclerectomy With Collagen Implant | JOURNAL OF GLAUCOMA | Bissig |
| 2009 | 68 | 4.7 | Retinal Prosthesis | Development of Microelectrode Arrays for Artificial Retinal Implants Using Liquid Crystal Polymers | INVESTIGATIVE OPHTHALMOLOGY & VISUAL SCIENCE | Lee |
| 2014 | 66 | 3.2 | Drug Delivery | Microneedle- mediated intrascleral delivery of in situ forming thermoresponsive implants for sustained ocular drug delivery | JOURNAL OF PHARMACY AND PHARMACOLOGY | Thakur |
| 2004 | 64 | 4.2 | Glaucoma | Results of the use of the Ex-PRESS miniature glaucoma implant in technically challenging, advanced glaucoma cases: a clinical pilot study | AMERICAN JOURNAL OF OPHTHALMOLOGY | Wamsley |
| 2008 | 64 | 3.5 | Glaucoma | Risk factors for delayed suprachoroidal haemorrhage following glaucoma surgery | BRITISH JOURNAL OF OPHTHALMOLOGY | Jeganathan |
| 2014 | 64 | 3.8 | Retinal Prosthesis | Laser patterning of platinum electrodes for safe neurostimulation | JOURNAL OF NEURAL ENGINEERING | Green |
| 2010 | 64 | 3.8 | Retinal Prosthesis | Evaluation of stimulus parameters and electrode geometry for an effective suprachoroidal retinal prosthesis | JOURNAL OF NEURAL ENGINEERING | Shivdasani |
| 2014 | 63 | 4.7 | Drug Delivery | Targeted Delivery of Antiglaucoma Drugs to the Supraciliary Space Using Microneedles | INVESTIGATIVE OPHTHALMOLOGY & VISUAL SCIENCE | Kim |
| 2014 | 63 | 4.7 | Retinal Prosthesis | Factors Affecting Perceptual Thresholds in a Suprachoroidal Retinal Prosthesis | INVESTIGATIVE OPHTHALMOLOGY & VISUAL SCIENCE | Shivdasani |
| 2012 | 63 | 3.8 | Retinal Prosthesis | Visual cortex responses to suprachoroidal electrical stimulation of the retina: effects of electrode return configuration | JOURNAL OF NEURAL ENGINEERING | Cicione |
| 2006 | 62 | 9.4 | Glaucoma | Aqueous shunts for glaucoma | COCHRANE DATABASE OF SYSTEMATIC REVIEWS | Minckler |
| 2013 | 62 | 3.2 | Glaucoma | Early postoperative safety and surgical outcomes after implantation of a suprachoroidal micro-stent for the treatment of open-angle glaucoma concomitant with cataract surgery | JOURNAL OF CATARACT AND REFRACTIVE SURGERY | Hoeh |
| 2018 | 62 | 2.6 | Glaucoma | Complications of micro-invasive glaucoma surgery | CURRENT OPINION IN OPHTHALMOLOGY | Yook |
| 2017 | 62 | 1.9 | Glaucoma | Safety and Efficacy of Microinvasive Glaucoma Surgery | JOURNAL OF OPHTHALMOLOGY | Chen |
| 2015 | 62 | 4.5 | Retinal Prosthesis | In Vivo and In Vitro Comparison of the Charge Injection Capacity of Platinum Macroelectrodes | IEEE TRANSACTIONS ON BIOMEDICAL ENGINEERING | Leung |
| 2008 | 62 | 2.3 | Retinal Prosthesis | A suprachoroidal electrical retinal stimulator design for long-term animal experiments and in vivo assessment of its feasibility and biocompatibility in rabbits | JOURNAL OF BIOMEDICINE AND BIOTECHNOLOGY | Zhou |
